# Computational insights into RNAi-based therapeutics for foot and mouth disease of *Bostaurus*

**DOI:** 10.1038/s41598-020-78541-6

**Published:** 2020-12-09

**Authors:** Tanmaya Kumar Sahu, Anoop Kishor Singh Gurjar, Prabina Kumar Meher, Cini Varghese, Sudeep Marwaha, Govind Pratap Rao, Anil Rai, Neha Guleria, Suresh H. Basagoudanavar, Aniket Sanyal, Atmakuri Ramakrishna Rao

**Affiliations:** 1 ICAR-Indian Agricultural Statistics Research Institute, New Delhi, India; 2 ICAR-Indian Agricultural Research Institute, New Delhi, India; 3 ICAR-Indian Veterinary Research Institute, Bengaluru, India; 4 Indian Council of Agricultural Research (ICAR), New Delhi, India

**Keywords:** Data processing, Pathogens

## Abstract

Foot-and-mouth disease (FMD) endangers a large number of livestock populations across the globe being a highly contagious viral infection in wild and domestic cloven-hoofed animals. It adversely affects the socioeconomic status of millions of households. Vaccination has been used to protect animals against FMD virus (FMDV) to some extent but the effectiveness of available vaccines has been decreased due to high genetic variability in the FMDV genome. Another key aspect that the current vaccines are not favored is they do not provide the ability to differentiate between infected and vaccinated animals. Thus, RNA interference (RNAi) being a potential strategy to control virus replication, has opened up a new avenue for controlling the viral transmission. Hence, an attempt has been made here to establish the role of RNAi in therapeutic developments for FMD by computationally identifying (i) microRNA (miRNA) targets in FMDV using target prediction algorithms, (ii) targetable genomic regions in FMDV based on their dissimilarity with the host genome and, (iii) plausible anti-FMDV miRNA-like simulated nucleotide sequences (SNSs). The results revealed 12 mature host miRNAs that have 284 targets in 98 distinct FMDV genomic sequences. Wet-lab validation for anti-FMDV properties of 8 host miRNAs was carried out and all were observed to confer variable magnitude of antiviral effect. In addition, 14 miRBase miRNAs were found with better target accessibility in FMDV than that of *Bos* *taurus.* Further, 8 putative targetable regions having sense strand properties of siRNAs were identified on FMDV genes that are highly dissimilar with the host genome. A total of 16 SNSs having > 90% identity with mature miRNAs were also identified that have targets in FMDV genes. The information generated from this study is populated at http://bioinformatics.iasri.res.in/fmdisc/ to cater the needs of biologists, veterinarians and animal scientists working on FMD.

## Introduction

Foot-and-mouth disease virus (FMDV) causes a highly infectious disease in domestic and wild cloven-hoofed animals^1^. Although, vaccination has been used to protect these animals against FMDV, there are shortcomings in the efficacy of available vaccines^1^. In such scenario, RNA interference (RNAi) appears as a promising strategy to control virus replication^2^. RNAi is triggered by small non-coding RNA molecules (ncRNAs) including short interfering RNAs (siRNA) and microRNAs (miRNA), and use of RNAi-based methods have been demonstrated as an alternative method of controlling the transmission of FMDV^3^. The antiviral power of short-hairpin RNAs (shRNA) or siRNAs against FMDV has been demonstrated widely by showing that RNAi based therapeutics can negatively impact the progression of FMDV infection. In particular, de los Santos et al*.*^4^ have demonstrated a significant reduction in the virus yield with a reduced viral RNA and protein synthesis as compared to the control cells by expressing an shRNA specific to 2B nonstructural protein coding region of FMDV RNA in porcine cells during the infection. They have also reported that their strategy is effective against multiple FMDV serotypes.

miRNAs are involved with the post-transcriptional regulation of gene expression and also demonstrated to play vital roles in the intricate interaction network between virus and host^5^. Chang et al*.*^6^ constructed multiple miRNAs targeting the Internal Ribosome Entry Site (IRES) of FMDV and found IRES-specific miRNAs significantly inhibited FMDV infection. They found that a co-cistronic expression plasmid containing two miRNA hairpin structures considerably postponed the deaths of suckling mice infected with lethal dose of FMDV vaccine strains of three serotypes (O, A and Asia 1). Du et al*.*^7^ induced the protection against FMDV in cell culture and transgenic suckling mice by miRNA targeting the integrin αv receptor. In the recent past, Samir and Pessler^8^ reviewed the association of small non-coding RNAs (ncRNAs) with viral infectious diseases. They presented an insight into the preclinical studies on using small ncRNAs to combat viral diseases affecting the livestock population. Many scientists across the globe explored the effectiveness of host miRNAs against FMDV infection. For instance, Gutkoska et al*.*^9^ have identified two host miRNAs i.e., miR-203a-3p and miR-203a-5p that impaired the FMDV infection across multiple FMDV serotypes. Moreover, artificial microRNAs (amiRs) can be seen as a new hope against viral diseases, if their limitations could be addressed successfully. Gismondi et al.^3^ discussed the structural implications of target selection while using amiRs as antiviral strategy to FMDV. Thus, these RNAi effectors have to be explored and evaluated extensively by focusing on the limitations of their applicability.

First line of defense against the viral infections is innate immune system and hence, utilization of the innate responses for antiviral, therapeutic and vaccine-adjuvation strategies is becoming an effective way for controlling these infections. Thus, looking at the successful use and possibility of FMD therapeutics development based on RNAi, this study was designed by focusing on identifying (i) targets of miRBase miRNAs in FMDV, (ii) miRNA targets in *Bos* *taurus* to uncover the miRNAs that target both host and pathogen, (iii) miRNAs of *Bos* *taurus* that target FMDV to study host RNAi based mechanisms against FMDV, (iv) targets of confirmed anti-FMDV miRNAs, (v) regions in FMDV genes that are not significantly similar with *Bos* *taurus* genes, as the probable targets for artificial siRNAs, (vi) targets of simulated nucleotide sequences (SNSs) in FMDV that do not have targets in *Bos* *taurus.* Subsequently, a database was populated with the integrated information obtained from the study.

## Materials and methods

### Data collection and processing

Sequence data required for studying the RNAi mechanism in FMD was collected from public resources.

#### Target sequences

FMDV and *Bos* *taurus* genomic sequences were retrieved from National Center for Biotechnology Information (NCBI) repository (https://www.ncbi.nlm.nih.gov/). The downloaded data contained 7991 FMDV sequences and 91,682 *Bos* *taurus* sequences. Additionally, 10,973 sequences of 1000 nucleotides upstream (1 K upstream) to the *Bos* *taurus* genes (contains the regulatory regions) were also downloaded from University of California, Santa Cruz (UCSC) Genome browser (https://genome.ucsc.edu/).

#### miRNA sequences

The mature miRNA sequences were downloaded from miRBase (release 21; http://www.mirbase.org/). miRBase contains published and annotated miRNA sequences having predicted hairpin portion of miRNA transcripts (termed as mir) along with the information on location and sequence of the mature miRNA (termed as miR)^10^. The downloaded sequence data from miRBase contained 35,828 mature miRNA sequences from 285 different organisms.

#### Redundancy removal

Homology reduction at 100% was carried out to remove identical sequences using CD-HIT server^11^ from all the sequence datasets i.e., mature miRNAs and target sequences of both FMDV and *Bos* *taurus*. The target nucleotide sequences from host and pathogen were subjected to homology reduction separately. The 1 K upstream regions containing 10,973 sequences were not subjected to homology reduction. The sequence data (before and after homology reduction) is shown in Table 1.

**Table 1:** Nucleotide sequence data before and after homology reduction.

Sequences	Before homology reduction	After homology reduction
FMDV	7991	6305
*Bos* *taurus*	91,682	80,709
mature miRNAs	35,828	21,426

### Tools used for target identification

As miRNA targets were to be identified in both *Bos* *taurus* and FMDV, tools that deal with various types of target genomes were sought. Most of the available tools for miRNA target prediction have few fixed animal and plant genomes. Hardly, there exists any tool to predict miRNA targets in viruses. However, psRNATarget^12^ has the flexibility to incorporate desired genome data apart from the already included plant genome data to identify targets of small RNAs. Though there are many differences exist between miRNAs of plants and animals, they do have significant amount of similarities. The miRNAs of both plants and animals are evolutionarily related and post-transcriptionally regulate gene expression through interactions with their target mRNAs^13^. Thus, psRNATarget was chosen as miRNA target identification tool in this study. However, to cross validate the psRNATarget results, another tool, GUUGle^14^ was used. In addition, the offline version of Basic Local Alignment Search Tool (BLAST)^15^ was used to identify the targetable regions on FMDV genome*.* These tools are described below in detail.

#### psRNATarget

psRNATarget, a plant small RNA target analysis server, which features two important analysis functions: (i) reverse complementary matching between small RNA and target transcript using an established scoring schema, and (ii) target-site accessibility evaluation by calculating the Un-Paired Energy (UPE) required to 'open' secondary structure around small RNA's target site on mRNA. It distinguishes translational and post-transcriptional inhibition, and reports the number of small RNA-target site pairs that may affect small RNA binding activity to target transcript. The psRNATarget searches target gene based on both complementarity scoring analysis and secondary structure analysis^12^.

#### GUUGle

*GUUGle* is an algorithm and a utility program that accepts a set of target sequences and a set of query sequences with a length threshold *k*. It reports all matches under RNA rules between the target and reverse complement of the query sequence having *k* or more consecutive base pairs^14^. GUUGle has been used here as a confirmatory tool to check whether at least one target is predicted for any miRNA whose targets have been predicted by psRNATarget. Thus, minimum number of targets has been set as 1 in GUUGle.

#### BLAST

The offline version of BLAST^15^ was used to identify the regions in FMDV genes that are not significantly similar with *Bos* *taurus* genes as the probable targets for artificial siRNAs.

### miRNA targets in FMDV and *Bos taurus*

The mature miRNA sequences were used as query to find their targets in FMDV and *Bos* *taurus* nucleotide sequence datasets. To achieve this, psRNATarget online server was used with default parameters and GUUGle was used offline with a command line user interface (CUI). The miRNAs not targeting any FMDV genes were discarded and remaining miRNAs were subjected to target identification in *Bos* *taurus.* The purpose was to identify the miRNAs having targets in FMDV but no targets or targets with higher UPE in *Bos* *taurus*. The UPE is generally used to evaluate mRNA site accessibility where lower energy values represent higher possibility to be an effective target site because the secondary structures may prevent small RNA and target site from contacting^12^.

### Identification and experimental validation of anti-FMDV miRNAs of *Bos taurus*

miRNAs of *Bos* *taurus* were isolated from the complete set of miRNAs collected from miRBase. Their targets in FMDV were identified using both psRNATarget and GUUGle to understand the built-in anti-FMDV mechanism in the host. The *Bos* *taurus* miRNAs identified to have targets in FMDV were subjected to experimental validation. The detailed experimental validation is elaborated below with appropriate sub-sections.

#### Maintenance of BHK-21 cells

The Baby Hamster Kidney 21 (BHK-21) cell line (ATCC), was maintained in Glasgow's Minimum Essential Medium (GMEM) (Himedia, India) supplemented with 10% Fetal Bovine Serum (FBS), 60 µg per milliliter (μg/ml) penicillin, 100 μg/ml streptomycin and 100 μg/ml kanamycin, in a humidified incubator with 5% CO_2_ at 37 °C.

#### Cellular toxicity test

To check the cellular toxicity of miRNA mimics on BHK-21 cells, different nanomolar (nM) concentrations of miRNA mimics (5, 10, 25 and 50 nM) were used and transfection was performed in a 24-well plate, using HiPerFect transfection reagent (Qiagen), according to the manufacturer’s protocol. The live cell percentage/growth was determined by trypan blue staining of cells at 24 and 48 h post transfection.

#### Optimizing concentrations of miRNA mimics for transfection

Four concentrations of negative miRNA mimics (10, 15, 20 and 25 nM) were selected for the transfection to assess non-specific effect of miRNA mimics on virus replication. FMDV O/IND/R2/75 was infected at 0.001 multiplicity of infection (MOI), at 24 h post transfection. Cell supernatant was harvested at 12 h post infection and virus titer was calculated by 50% Tissue Culture Infectious Dose (TCID_50_) assay in BHK-21 cells^16^.

#### Transfection of miRNA mimics

The mimics of the considered *Bos* *taurus* miRNAs were custom prepared using Qiagen technology and the negative control miRCURY LNA miRNA mimic (UCACCGGGUGUAAAUCAGCUUG; not targeting either FMDV or any known vertebrate gene) from Qiagen (Cat#YM00479902) was used as the control. BHK-21 cells were transfected with miRNA mimics diluted to 10 nM in respective wells of a 24-well plate along with a mock control using HiPerFect transfection reagent (Qiagen), according to the manufacturer’s protocol. Further, to assess the antiviral activity of the miRNA mimics, FMDV O/IND/R2/75 was infected with 0.001 MOI, at 24 h post transfection. Cell supernatant was harvested at 12 h post infection and viral growth was monitored by titrating the progeny virus in the supernatant of the infected cell culture by TCID_50_ assay.

#### *TCID*_*50*_*assay*

TCID_50_/ml was determined from the cellular supernatant, to estimate the amount of progeny virus. Initially, 50 micro liter (μl) of tenfold diluted virus was added in quadruplet well containing 50 μl GMEM medium in 96-well cell culture plate. Then 50 μl (50,000 cells/well) of a suspension of BHK-21 cells was added. The cell plates were incubated at 37 °C under 5% CO_2_ conditions for 48 h. After incubation, each well was observed for the cytopathic effect (CPE) and TCID_50_/ml was calculated^16^.

#### Statistical analysis

Statistical analysis was carried out on the data obtained from wet-lab experiments that were repeated for three times. All values are expressed as "mean ± standard deviation". The difference between treatment means was tested using Student’s t-test to assess the statistical significance (p-value less than 0.05 was considered as significant difference between treatment means).

### Prediction of plausible targets of confirmed anti-FMDV miRNAs

The information on anti-FMDV miRNAs were collected from VIRmiRNA^17^ database that is a resource for experimentally validated viral and anti-viral miRNAs with their corresponding targets. These miRNAs do not have any corresponding miRBase accessions. In addition to the reported targets, other plausible targets in FMDV were also predicted for these miRNAs using psRNATarget and GUUGle with default set of parameters.

### Detection of targetable regions in FMDV

siRNAs seek perfect complementarity with the targets unlike miRNAs to knock down specific genes, with minor off-target exceptions^18^. Though, both miRNA and siRNA share same processing machinery, nature of the regulatory target is different for both. As miRNAs do not require exquisite sequence complementarity, they can impact the expression of multiple genes, not just similar ones unlike siRNAs. Further, the degree of sequence complementarity between miRNA and its intended target mRNA can have differential outcomes. Therefore, few biotechnology companies are currently focusing on siRNA-based treatments for various diseases along with other RNAi based therapeutics^19^. Various siRNAs have been designed against the gene encoding the VP1 protein of FMDV to inhibit the viral replication^20–22^. However, it is also necessary to consider that the designed siRNA should not have any off-target effect on the host genes. Of course, the evaluation of siRNAs for causing cytotoxicity to the cells has to be carried out before its practical application. In the light of above facts, we tried to identify the FMDV genes, which can be targeted by exogenous siRNAs, with the possible target regions that does not have significant similarity with any part of the host genes.

Initially, all FMDV sequences were locally aligned with the *Bos* *taurus* sequences using the standalone NCBI BLAST application. Genomic sequences of *Bos* *taurus* were set as a local database for the standalone BLAST whereas the FMDV sequences were subjected to similarity search against this local database. The FMDV sequences having no significant similarity with *Bos* *taurus* sequences were filtered out and split into fragments of 23 nucleotides that is equivalent to the length of dicer-processed siRNA i.e., 21–23 nucleotides^23^. Further, these gene fragments were realigned with *Bos* *taurus* sequences using the offline BLAST by adjusting the parameters for short nucleotide sequences. Finally, coordinates of the gene fragments which showed no hits against host sequences were reported as targetable regions of the corresponding FMDV genes. The target sequences are identical to sense strand of the respective siRNAs. Hence, properties like GC content^24^, melting temperature^25,26^, positional occurrence or non-occurrence of certain nucleotides in the sense strand^27,28^ responsible for siRNA activity were also computed for each targetable region. Positional preferences of specific nucleotides in the siRNA sense strand is well described in Zhou et al.^27^ and Reynolds et al.^28^. They suggested that occurrence of A at 3rd position, T at 10th position, T at 13th position, G or C at first position and A or T at 19th position and non-occurrence of G at 13th position on the siRNA sense strand is vital for potential siRNA selection, as these sense strand base preferences improve siRNA functionality.

### Targets of SNSs in FMDV

SNSs are the nucleotide sequences of length 25 bases, created computationally from four nucleotide bases (A, T, G and C) by simple random sampling with replacement. The R-code used for generating a set of 150,000 SNSs is provided as Supplementary Fig. 1. SNSs containing the nucleotide repeats of length > 4 bases were discarded to avoid low complexity regions. Then targets of the remaining SNSs in FMDV were identified using GUUGle. The sequences targeting FMDV genes were subjected to target identification against *Bos* *taurus* genes. SNSs having targets only in FMDV and not in *Bos* *taurus* were selected and aligned against all downloaded mature miRNAs of miRBase using the offline BLAST program. SNSs having a high similarity with established set of miRNAs showed their possible miRNA-like properties.

### Development of database

**Figure 1:**
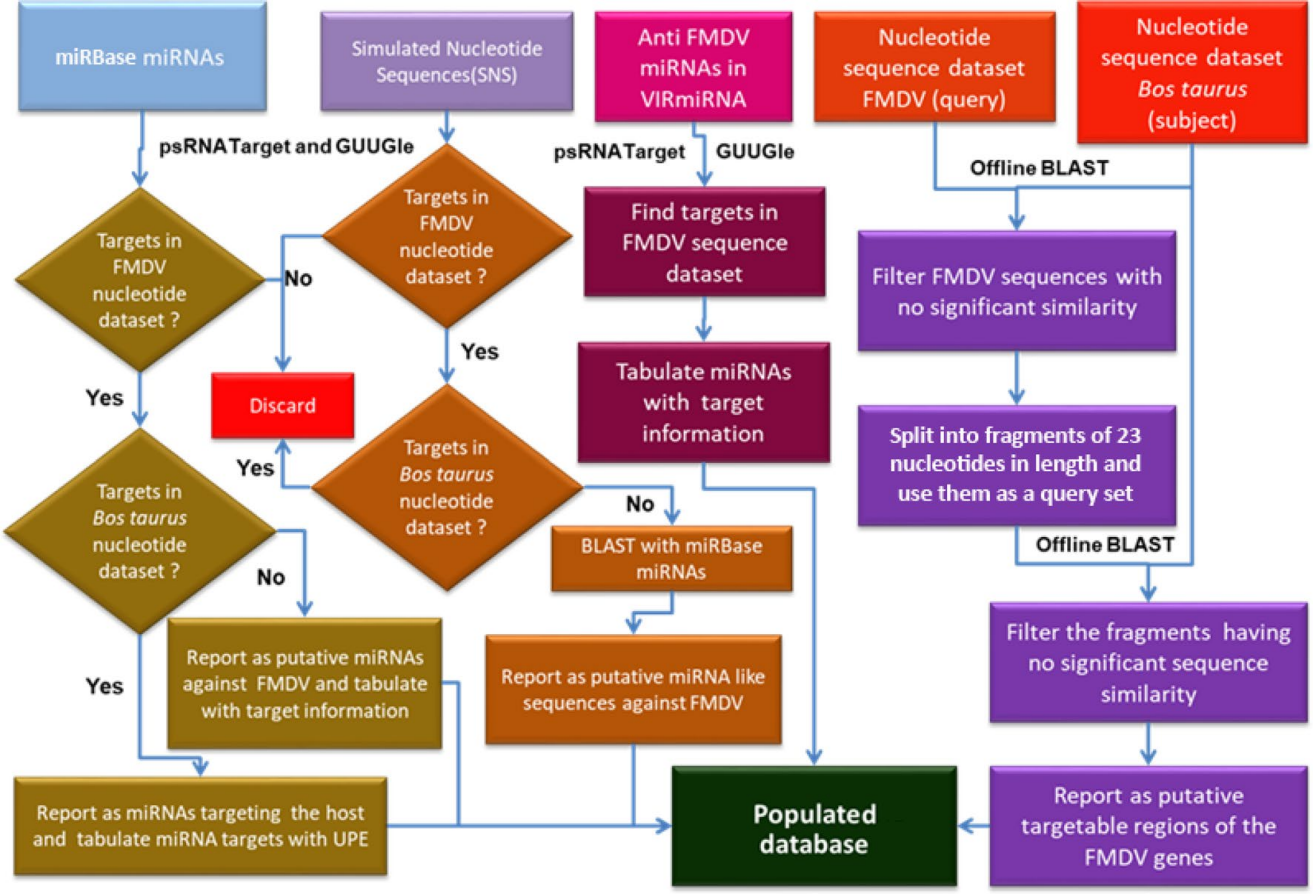
A flow diagram showing identification of anti-FMDV miRNAs and development of a database of miRNAs related to FMD along with their targets.

A collection of putative anti-FMDV miRNAs, SNSs (artificial miRNA like sequences) and probable targetable regions on FMDV genes were tabulated and stored in a MySQL database. A user friendly web interface for searching RNAi related information was created as part of "Foot and Mouth Disease Information System for Cattle (FMDISC)" with a three layered architecture. The Hypertext Markup Language (HTML) and Cascading Style Sheets (CSS) have been used as client side interface layer (front-end). The Personal Home Page: Hypertext Preprocessor (PHP) has been used as the server side application layer (middle Layer). The MySQL served as the database layer at the back end. Java script was used for client side customizations. A detailed flow diagram on identification of anti-FMDV miRNAs and development of the database on miRNAs related to FMD along with their targets is presented in the Fig. 1.

## Results

The targets of miRBase miRNAs in FMDV and *Bos* *taurus* were predicted by using both psRNATarget and GUUGle. From the VIRmiRNA database, 4 anti-FMDV miRNAs were collected and examined in silico for their other possible targets in FMDV. The regions on FMDV genes were located that are not significantly similar with *Bos* *taurus* genes. These regions are expected to serve as the putative targets for artificial siRNAs. A number of SNSs that share similarity with miRBase miRNAs were also proposed here as the possible silencing agents for FMDV genes which can be further validated. The information obtained from this in silico analysis was documented and populated in the form of a database.

### miRNAs targets in host and pathogen

Out of 35,828 miRNAs of miRBase, possible targets of 641 and 438 miRNA sequences in FMDV were identified through psRNATarget and GUUGle respectively (Table 2). For these miRNAs, more targets were observed in *Bos* *taurus* than in FMDV*.* Thus, a comparison of miRNA targets was made in host and pathogen based on UPE. In this context, 116 miRNAs were found with lesser UPE in FMDV than *Bos* *taurus*. Table 3 shows 14 miRNA accessions for which the UPE for FMDV targets is at least 5 units lesser than that of *Bos* *taurus* targets. The miRNA accession MIMAT0031768 targeting the FMDV accession FV536933.1 was observed with highest UPE difference of 10 units than its target in *Bos* *taurus*. It is also noticed that out of the 14 targets in *Bos* *taurus*, 7 are predicted sequences.

**Table 2:** Number of targets of miRNAs in FMDV and *Bos* *taurus* predicted by GUUGle and psRNATarget.

Serial number	No of miRNA accessions	No of target accessions	Target species	Tool
1	438	135	FMDV	GUUGle
2	641	3127	FMDV	psRNATarget
3	438	425	*Bos* *taurus*	GUUGle
4	641	41,340	*Bos* *taurus*	psRNATarget

**Table 3:** miRNA accessions for which the UPE for FMDV targets is at least 5 units lower than that of *Bos* *taurus* targets.

miRNA accessions	FMDV target accessions	FMDV UPE	*Bos* *taurus *target accessions	*Bos* *taurus* UPE	UPE difference
MIMAT0031768	FV536933.1	8	NM_001034053.1	18	10
MIMAT0030066	FV536917.1	8	XM_002693816.3	16	8
MIMAT0021045	FV536917.1	6	XM_002698456.5	13	7
MIMAT0019151	FV536931.1	6	XM_015458263.1	13	7
MIMAT0021579	FV536913.1	8	NM_001191430.1	15	7
MIMAT0018266	FV536925.1	6	NM_001046526.2	12	6
MIMAT0031743	FV536915.1	8	XM_015472936.1	14	6
MIMAT0026403	FV536929.1	8	XM_010813729.2	14	6
MIMAT0004312	FV536929.1	5	NM_174265.2	10	5
MIMAT0024773	FV536915.1	6	NM_001097992.2	11	5
MIMAT0005101	FV536931.1	6	XM_001256435.1	11	5
MIMAT0025620	FV536913.1	7	XM_005226869.3	12	5
MIMAT0017951	FV536925.1	8	NM_001102076.2	13	5
MIMAT0007393	FV536927.1	9	NM_001038177.1	14	5

### miRNAs of *Bos taurus* targeting FMDV genes

The miRNAs of *Bos* *taurus* from miRBase and VIRmiRNA were examined for their targets in FMDV to understand the probable host RNAi mechanism against FMD. A total of 284 targets (constituting the union of targets predicted by psRNATarget or GUUGle) in 98 distinct FMDV genomic sequences were identified for 8 mature miRNAs from miRBase and 4 from VIRmiRNA. Table 4 represents the number of FMDV targets of *Bos* *taurus* miRNAs. The highest number of targets has been predicted for the miRNA accession MIMAT0011924 for which GUUGle has also predicted one target. Both GUUGle and psRNATarget predicted same target (FV536931.1) for the miRNA accession MIMAT0024589, where the target region on the FV536931.1 also coincide (4572–4594 for psRNATarget and target start is 4574 in GUUGle).

**Table 4:** Number of targets predicted by psRNATarget and GUUGle in FMDV for *Bos* *taurus* miRNAs.

miRNA accession	miRNA symbol	psRNATarget	GUUGle^a^	Total number of targets
MIMAT0011794	miR-2287	3	1	4
MIMAT0011867	miR-2334	46	0	46
MIMAT0011904	miR-2366	1	1	2
MIMAT0011924	miR-2378	96	1	97
MIMAT0011955	miR-2399-3p	14	1	15
MIMAT0011966	miR-2408	7	1	8
MIMAT0013852	miR-2894	1	1	2
MIMAT0024589	miR-6119-3p	9	1	10
AVM_004	miR-3D-1311	41	1	42
AVM_005	miR-3D-657	13	1	14
AVM_006	miR-3D-715	38	1	39
AVM_007	miR-3D-983	15	1	16

^a^GUUGle was used as a confirmatory tool to check whether at least one target is predicted by this tool for the miRNA whose targets have been predicted by psRNATarget.

### Antiviral response of *Bos taurus* miRNAs against FMDV

Cellular toxicity was observed in the cells when transfected at 25 nM and higher miRNA mimic concentrations after 24 and 48 h, whereas at 5 and 10 nM the cell growth pattern was at par with mock (Supplementary Fig. 2a). This suggests that there is a limit to the miRNA mimic concentration that can be transfected into BHK-21 cells. Virus titration was done which also showed that higher mimic concentrations have significant inhibition effect against the virus in comparison to mock control (Supplementary Fig. 2b). Further, eight test miRNAs transfected at 10 nM concentration did not show cellular toxicity (Supplementary Fig. 2c). Therefore, 10 nM mimic concentration was used for actual transfection of the miRNAs.

**Figure 2:**
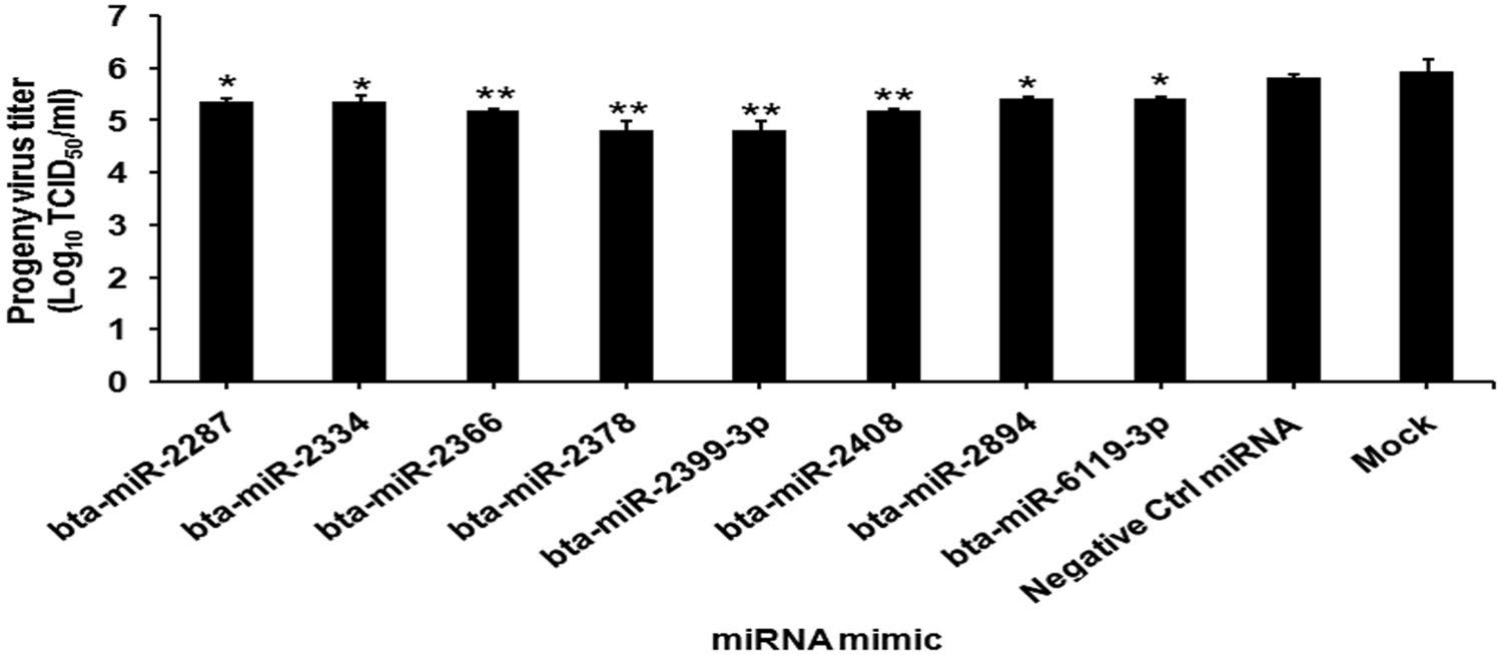
The FMDV progeny yield (TCID_50_/ml) at 12 h post infection in BHK-21 cells transfected with 8 selected miRNAs in comparison with a negative control miRNA and mock control. The results are expressed as the "mean ± standard deviation" from three independent experiments, *p < 0.05, **p < 0.01.

The miRNA mimics of 8 miRNAs viz., bta-miR-2287, bta-miR-2334, bta-miR-2366, bta-miR-2378, bta-miR-2399-3p, bta-miR-2408, bta-miR-2894, and bta-miR-6119-3p that were expressed transiently in BHK-21 cells and infected with FMDV showed variable antiviral effect, as indicated by altered progeny virus titer compared to control miRNA mimic. We observed significant reduction in the progeny virus titer in the culture supernatant at 12 h post infection where the predicted miRNAs possibly have restricted the viral replication to a varying extent by targeting different regions of invading FMD virus (Fig. 2). Compared to the negative control miRNA and mock control, bta-miR-2366, bta-miR-2378 bta-miR-2399-3p and bta-miR-2408 showed 1 Log_10_ virus reduction in BHK-21 cells.

### Identification of targetable regions in FMDV

FMDV accessions along with their sequence coordinates were identified having no significant similarity with the *Bos* *taurus* genes. A total of 530 FMDV accessions were identified to have no significant similarity with the *Bos* *taurus* accessions. To accurately identify the targetable regions on these 530 FMDV accessions, their sequences were split into 23 nucleotides length fragments. The splitting resulted in 25,213 fragments, out of which only 3279 fragments were identified with no significant similarity when realigned to the *Bos* *taurus* sequences**.** Out of 3279 fragments, only 8 fragments were obtained with all favorable positional occurrences of nucleotides (A at 3rd position, T at 10th position, G not at 13th position, T at 13th position, G or C at first position and A or T at 19th position)^24–28^ in the sense strand of siRNA that is identical to the target region (Table 5). The mapping coordinates of these fragments on the respective FMDV genes were reported as the putative targetable regions.

**Table 5:** Targetable regions of FMDV accessions having all favorable positional occurrences/non-occurrences of nucleotides.

Target accession	Target description	Target coordinate	GC (%)	TM (°C)
KJ831736.1	FMDV-O isolate BRA/4/94 capsid protein gene	1956–1978	43.478	53.491
KJ831741.1	FMDV-O isolate O/BRA/01/1992 capsid protein gene	1956–1978	43.478	53.491
KJ831743.1	FMDV-O isolate O/BRA/02/1994 capsid protein gene	1956–1978	43.478	53.491
KJ831744.1	FMDV-O isolate O/BRA/03/1994 capsid protein gene	1956–1978	43.478	53.491
KJ831746.1	FMDV-O isolate O/BRA/06/1994 capsid protein gene	1956–1978	43.478	53.491
KJ831747.1	FMDV-O isolate O/BRA/08/1994 capsid protein gene	1956–1978	43.478	53.491
KJ206910.1	FMDV-O isolate SAU/3/2013 polyprotein gene	484–506	43.478	53.491
HM067705.1	FMDV-SAT2 isolate Buffalo 10 QE polyprotein gene	5797–5819	65.217	62.404

The first 6 FMDV accessions (Table 5) are highly similar at the target regions that are from different isolates of same serotype (FMDV-type O). Thus, they have identical sequences (GTACAACGGTAGTTGCAGATACA) as well as same target coordinates, GC content and melting temperature. The seventh accession is also from the same serotype that have same GC content and melting temperature but different target coordinates. The last accession HM067705.1 (GTAACCCCGTTGTCGGCGTGGTC) is a polyprotein gene of FMDV-SAT2. Though only 8 fragments (targetable regions) have shown favorable properties, all the 3279 fragments (having no significant similarity with host genome) were populated as probable targetable regions in the database along with the properties like GC content, melting temperature, positional occurrence or non-occurrence of certain nucleotides.

### Targets of SNSs in FMDV

Apart from publicly available natural miRNAs, artificial miRNAs also have been reported to confer antiviral effect^3^. Thus, identifying artificial miRNAs or miRNA-like agents through in silico sequence simulation and target prediction approaches may provide novel insights into FMD therapeutics. With this expectation, we have randomly generated 150,000 SNSs of length 25 nucleotides, out of which, only 60 SNSs (Supplementary Table 1) were found to have targets only in FMDV and not in *Bos* *taurus*. These 60 SNSs have 60 targets in 12 distinct FMDV accessions. Sixteen out of these 60 SNSs were observed to have similarity with 41 mature miRNAs. The maximum and minimum values for alignment length, identity and bit score are presented in Table 6.

**Table 6:** Summary of the similarity between 16 SNS and miRBase miRNAs.

Alignment length	Value	Query (number of SNSs)	Subject (number of miRNAs)	Identity (%)	Bit score^a^
Max	17	1	1	94.12	22.9
Min	11	15	25	100	21.1

Identity	Value (%)	Query (number of SNSs)	Subject (number of miRNAs)	Alignment length	Bit score
Max	100	15	37	11–12	21.1–22.9
Min	92.86	4	4	14	21.1

Bit score	Value	Query (number of SNSs)	Subject (number of miRNAs)	Alignment length	Identity (%)
Max	22.9	9	11	12–17	94.12–100
Min	21.1	15	29	11–14	92.86–100

^a^A high bit score indicates a better alignment.

The SNS sim_21145 (Supplementary Table 1) have shown maximum alignment length with the miRNA miR7516-5p possessing highest bit score and lowest e-value (0.31). The lower e-value indicates a higher significance of sequence similarity whereas a higher bit score designates a better sequence similarity^15^. These two scores together are used to compare the pair-wise alignments from different similarity searches. The SNSs having a high identity with the established set of miRBase miRNAs are reported in the study as miRNA like SNS and populated in the FMDISC.

### Anti-FMDV repository

The anti-FMDV repository can be accessed from "miRNA" section of FMDISC (http://bioinformatics.iasri.res.in/fmdisc/) that has been developed and maintained at ICAR-Indian Agricultural Statistics Research Institute, New Delhi, India. The repository is enriched with the information on anti-FMDV miRNAs, putative targetable regions on FMDV genes, miRNA like SNSs with their targets in FMDV. The user friendly web interface provides appropriate search options for the miRNA of interest related to FMD. Web interfaces to search the information regarding anti-FMDV miRNAs, targetable regions on FMDV genomic sequences and FMDV targets of putative miRNA-like-SNS are described below with the appropriate snapshots.

#### miRNA targets on FMDV

**Figure 3:**
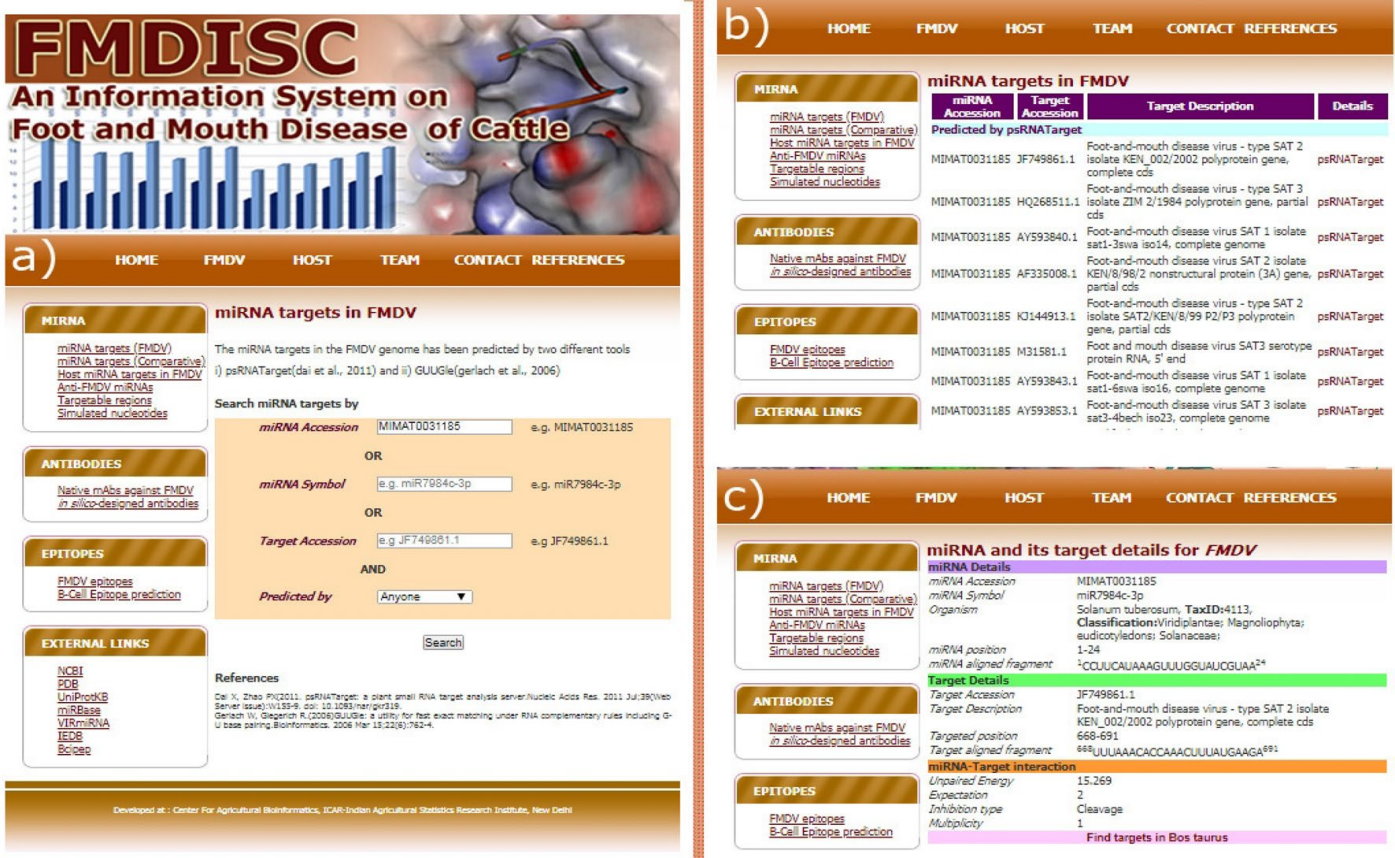
Interface for miRNA target search in FMDV showing (**a**) search interface, (**b**) listing of miRNAs based on search parameters and (**c**) miRNA and its target details.

The search interface for miRBase miRNA targets in FMDV provides the capability to search based on miRBase miRNA accession, miRNA symbol and FMDV target accession. It also provides interface for displaying the predicted targets by four options; (i) psRNATarget, (ii) GUUGle, (iii) Both, and (iv) Anyone (Fig. 3). By selecting “psRNATarget”, the targets only predicted by psRNATarget are displayed. Similarly, the targets predicted only by GUUGle are displayed upon selecting “GUUGle”. When the user selects “Both” or “Anyone”, intersection or union of the targets predicted by the above mentioned tools is displayed respectively.

The search interface for miRNA targets in FMDV is shown in Fig. 3a. The search results containing the list of miRNAs based on the search parameters is displayed in the Fig. 3b. The details on the miRNA of interest selected from the list (Fig. 3b) and its target are shown in the Fig. 3c with a link for finding its target in *Bos* *taurus*. The targets in *Bos* *taurus* are shown in the similar interface as displayed in the Fig. 3b,c.

#### miRNA targets compared in both host and pathogen based on UPE

**Figure 4:**
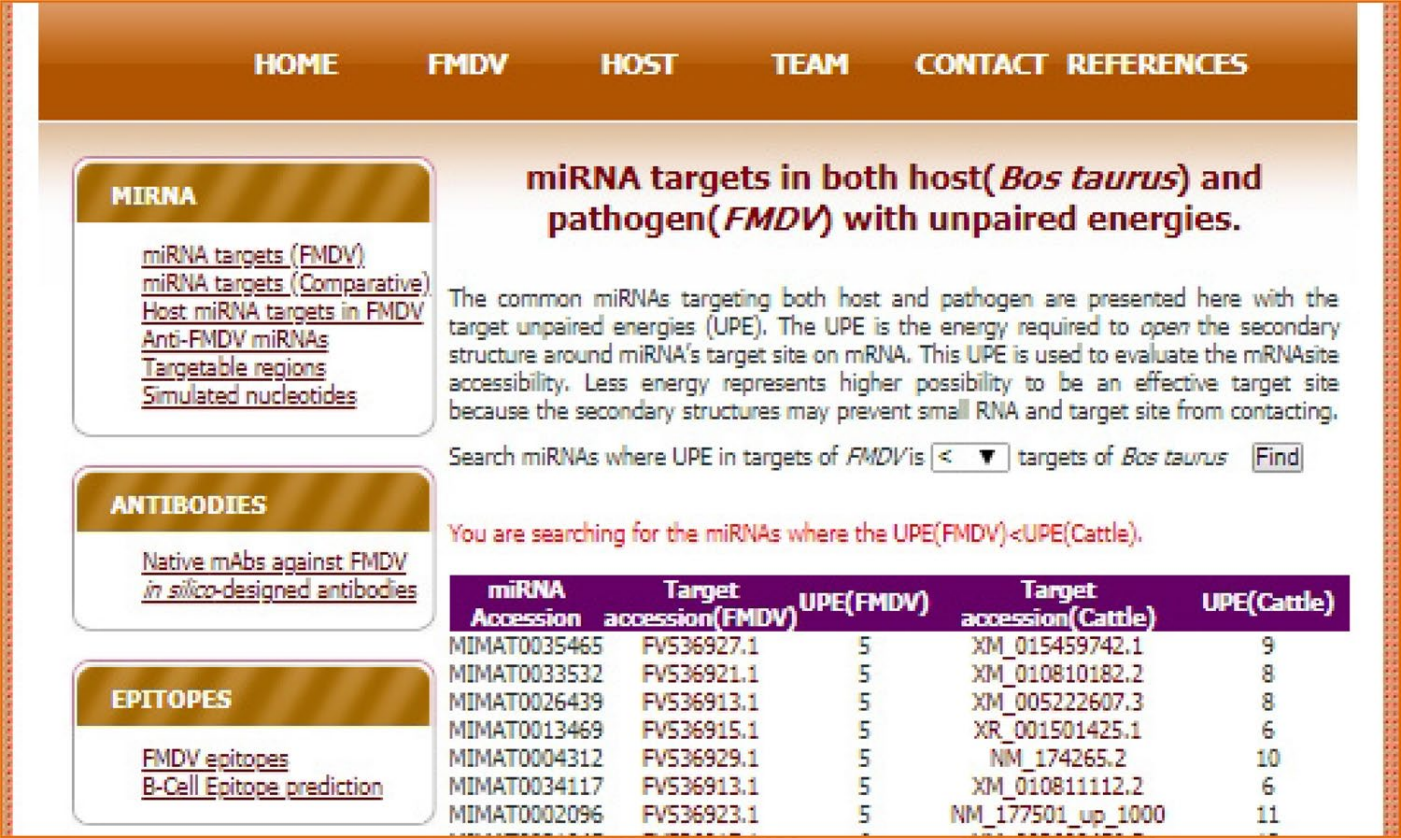
Comparison between FMDV and *Bos* *taurus* miRNA targets based on UPE, where a lower UPE represents a better target accessibility.

This section of the repository gives information on the miRNAs that target both FMDV and *Bos* *taurus* with their corresponding UPEs. The details of these targets can be viewed by clicking on the respective target accessions. As a lower UPE represents a better target accessibility, the miRNAs with low UPE in FMDV and high UPE in *Bos* *taurus* may have vital importance in therapeutic developments for FMD. The snapshot of search interface for miRNAs targeting both host and pathogen is presented in the Fig. 4 with target UPE.

### Host miRNA (miRBase and VIRmiRNA) targets in FMDV

**Figure 5:**
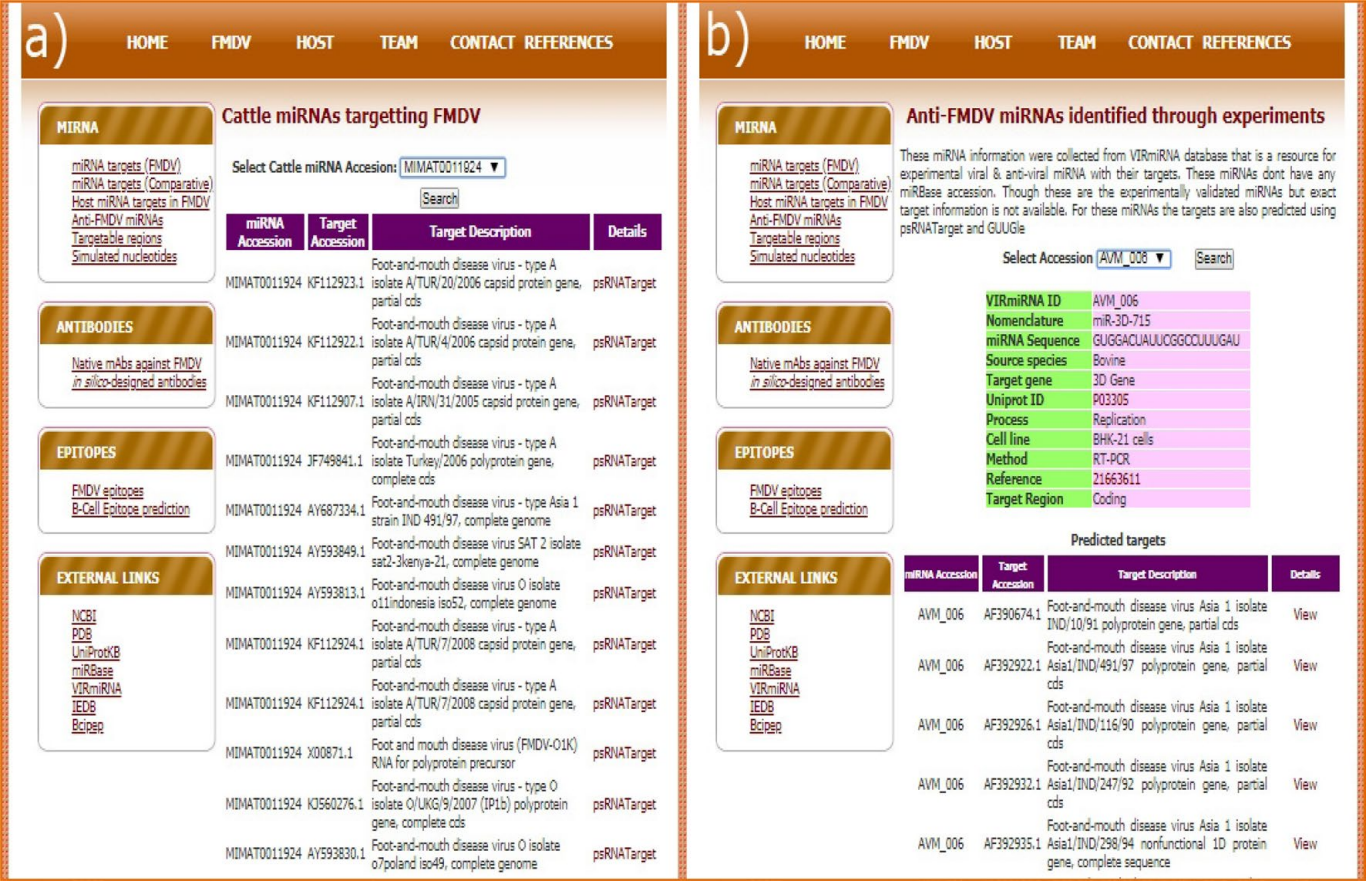
Search interface for host miRNA targets for (**a**) miRBase and (**b**) VIRmiRNA anti-FMDV miRNAs.

A separate search interface was designed specifically for the cattle miRNAs targeting FMDV genes. The host miRNAs includes 8 miRNAs from miRBase and 4 from VIRmiRNA database. The miRNAs of VIRmiRNA database are the confirmed anti-FMDV miRNAs. The search interface (Fig. 5a) is designed with the option to select the host miRNAs which results a list of targets for the selected miRNA. For miRBase miRNAs, the target details are provided in a similar fashion as shown in Fig. 3c. The targets for miRNAs of VIRmiRNA database can be searched from both the links "*Host* *miRNA* *Targets* *in* *FMDV*" and "*Anti-FMDV* *miRNAs*". In the later one, additional details on the process, cell line, method of detection are given as shown in Fig. 5b.

### Targetable regions

**Figure 6:**
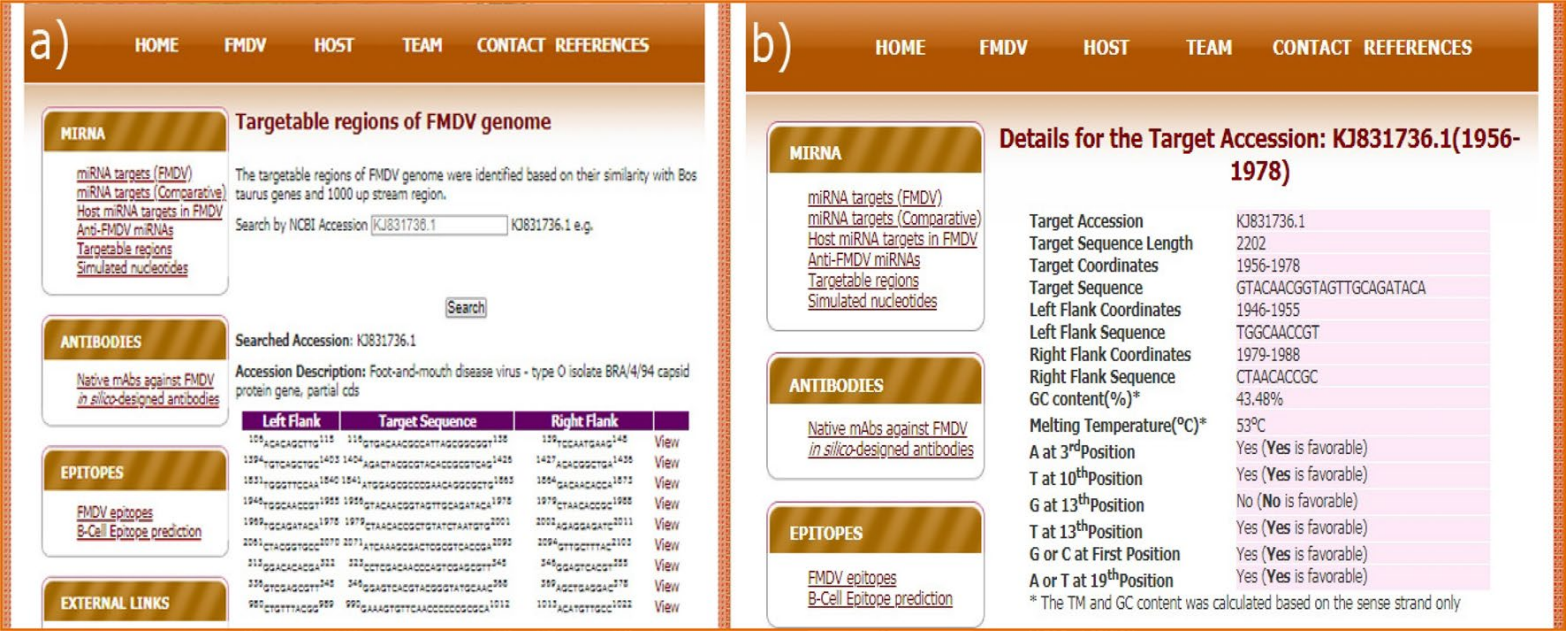
(**a**) Search interface and (**b**) result page for the putative targetable regions on the FMDV accessions.

The targetable regions on the FMDV accessions can be searched by selecting an accession from the list given in search interface (Fig. 6a). The details of the targetable regions including the target accession, sequence of the targetable region, coordinates, left and right flanking sequences, GC content, melting temperature, positional occurrence/non-occurrence of nucleotides are provided in the result page (Fig. 6b).

### Simulated nucleotides

**Figure 7:**
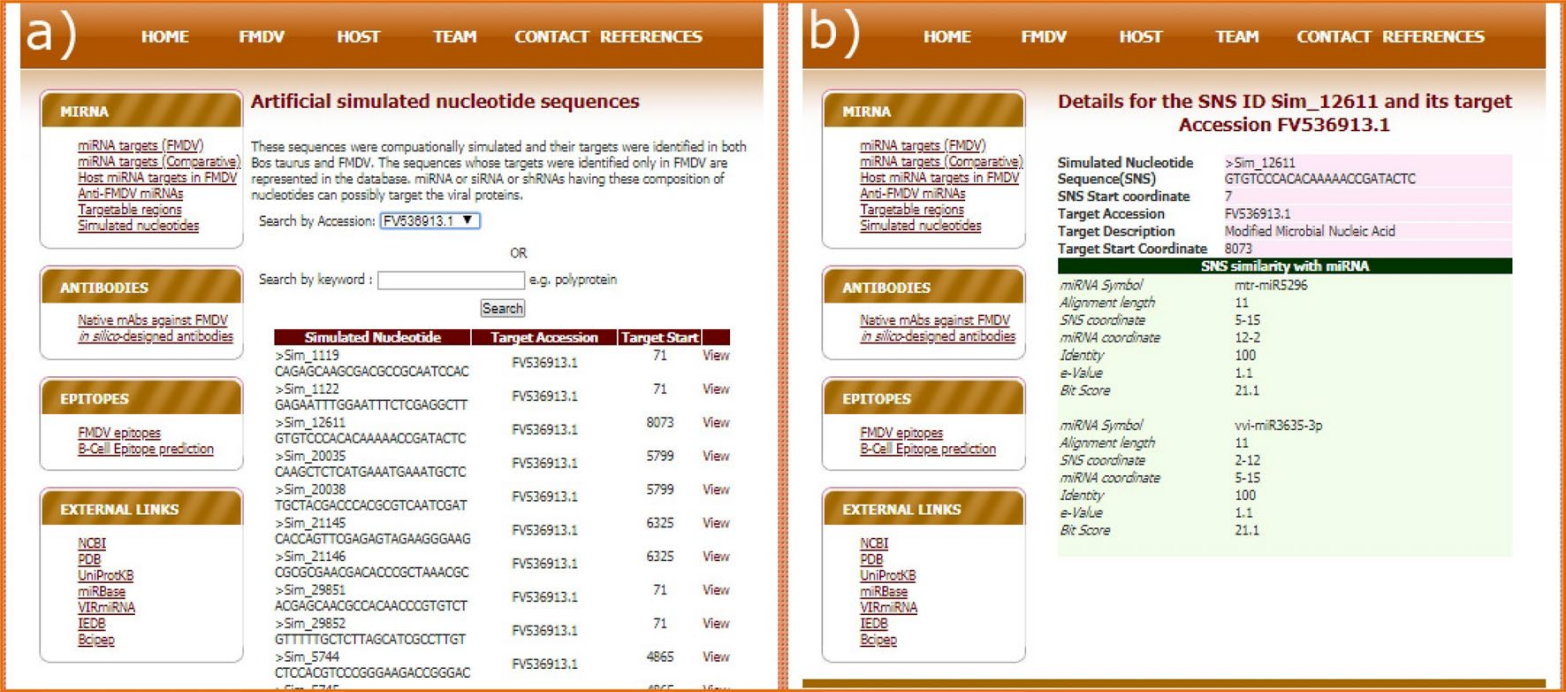
(**a**) Search interface and (**b**) result page of SNS targets in FMDV. The result page shows the SNS details, target details and the similarity of the SNS with miRBase miRNAs.

The targets of SNSs can be searched by selecting the FMDV accessions from the list or by searching a keyword through the text box provided in the search interface (Fig. 7a). The results page shows a list of SNS with their corresponding targets for a particular search. Clicking on the "View" link against each SNS takes the user to another page where details of SNS, its target and its identity with miRBase miRNAs are provided (Fig. 7b).

## Discussion

RNAi based therapeutics present a new strategy for inhibiting the replication of certain viruses^2^. Specifically, antiviral miRNAs and siRNAs against FMDV have demonstrated an alternative method of slowing down the viral transmission^3^. Thus, in silico approaches were explored in this study to understand applicability of RNAi based therapeutic solutions for FMD. Based on the available resources and cell line, a handful of host miRNAs were also experimentally validated at ICAR-Indian Veterinary Research Institute, Bangalore, India. Computationally, miRNA targets in both host and pathogen have been identified in this study as well as anti-FMDV miRNAs in the host have also been explored in silico and evaluated experimentally. The number of miRNA targets identified for *Bos* *taurus* is much more than the targets identified in FMDV because FMDV genome size is very small in comparison to *Bos* *taurus* genome. The FMDV genome ranges from 7000 to 8500 bp and varies depending on serotypes^29^ whereas the size of bovine genome is ~ 2.5 Gbp^30^ that is ~ 300,000 times higher than FMDV genome. The study of anti-FMDV miRNAs in *Bos* *taurus* revealed 12 mature miRNAs (8 from miRBase and 4 from VIRmiRNA databases) having a total of 284 targets in 98 distinct FMDV genomic sequences, which suggests the possible existence of miRNA based anti-FMDV mechanism in *Bos* *taurus* to defend the FMDV.

Viable miRNA targets have been identified by researchers worldwide with high reliability through miRNA profiling efforts that have been conducted with FMDV infected pigs and cattle^31,32^. This has inspired us to experimentally validate a handful of host miRNAs for their anti-FMDV properties. As 4 host miRNAs from VIRmiRNA database were reported to be anti-FMDV miRNAs, only the remaining 8 host miRNAs from miRBase were experimentally validated. The validation results showed that the mimics of these miRNAs targeted different genomic regions of the invading FMD virus and restricted its replication. The negative control miRNA mimic did not impact the viral replication. However, the magnitude of the antiviral effect of the 8 miRNA mimics was observed to be varying. This could be attributed to the difference in the degree of sequence-specific miRNA interactions with viral elements as reported previously^31,33^ and depends on the concentrations of miRNAs and targets. Further, the results of the experimental validation of all the considered 8 host miRNAs also supported the in silico predictions by more or less reducing the viral replication*.* However, further investigation is required to evaluate the efficacy of these miRNAs in vivo for establishing their potentiality in the development of novel therapeutic interventions during the FMDV infection.

The comparison of miRNA targets in host and pathogen based on UPE revealed 14 miRNA accessions for which the UPE for FMDV targets is at least 5 units lesser than that of *Bos* *taurus* targets. A lesser UPE represents higher possibility to be an effective target site^12^. Thus, these 14 miRNAs may be subjected to experimental validation in the context of FMDV therapeutics. In addition, the miRNA MIMAT0031768 targeting the FMDV accession FV536933.1 was observed with highest UPE difference of 10 units then its target in *Bos* *taurus*. The FV536933.1 is an 8170 bp long linear DNA of FMDV defined as "modified microbial nucleic acid"*.* The sequence of a "modified microbial nucleic acid" constitute 3 out of 4 nucleotide bases and does not contain methylated or other cytosine alterations, instead, all cytosines are converted to uracil. Online BLAST results revealed that FV536933.1 shares more than 70% identity at 99% query coverage with 4 different strains of FMDV serotype SAT3 genomic sequences (AY593850.1, MG372727.1, KR108950.1 and KM268901.1) where the target regions (3843.0.3866) belong to the 1D (VP1) gene (3220.0.3873). It is worth mentioning that VP1 is one of the surface protein on the FMD virus particle that has an important role in determining virus antigenicity and receptor-mediated entry into the host cell^34^.

The genome comparison of both host and pathogen revealed only 530 out of 6305 FMDV sequences are not significantly similar with that of *Bos* *taurus.* Probably, this could be the reason for obtaining a good number of miRNAs having targets in both FMDV and *Bos* *taurus.* In order to identify targetable regions in FMDV genomic sequences, these 530 FMDV sequences were split into fragments of 23 nucleotides in length that resulted in 25,213 subsequences. Only 3279 out of 25,213 subsequences were found to be dissimilar with the *Bos* *taurus* genomic sequences. Further, only 8 out of 3279 subsequences were obtained with all favorable positional occurrences of nucleotides as in the sense strand (identical to the target region). The coordinates of these fragments are noticed to be mapped with the capsid gene of FMDV serotype O and polyprotein genes of both FMDV serotypes O and SAT2. The experimentally validated targetable regions are expected to contribute in the development of RNAi based therapeutics for FMD of cattle.

The main purpose of constructing a number of SNSs and finding their corresponding targets in FMDV was to identify anti*-*FMDV miRNA-like nucleotide fragments sharing similarity to miRBase miRNAs. Out of 150,000, only 16 SNS were observed to have targets in FMDV being > 90% identical with 41 mature miRNAs. The identity summary given in Table 6 confirms the homology of such SNS with the established set of miRNAs. Thus, miRNAs or siRNAs based on these artificial nucleotide sequences are expected to potentially contribute to the management of FMD, but must be successfully validated both in vitro and in vivo.

RNAi approach has been widely used for drug development and several phase I and II clinical trials are under way^35^. Despite enthusiastic studies in a large number of animal models, including systemic delivery to non-human primates^36^ there are a number of obstacles and concerns that remain. Some of these hurdles include but are not limited to: off-target effects, triggering of type I interferon responses, and an effective means of in vivo delivery. These issues need to be resolved before widespread application of RNAi-based therapeutics^37^. In addition, the success rate of therapeutic applications of siRNAs in both in vivo and in vitro conditions depends on various factors like, size of siRNA molecule, use of appropriate vectors and many more. Specifically, the effectiveness of RNAi as an antiviral strategy against RNA viruses like FMD encompasses additional limitations like emergence of resistant mutants, viral suppressors, cytotoxicity, high genetic variability of FMDV, large number of susceptible animals and economical feasibility^38,39^. To overcome these hurdles, multiple delivery vehicles are now in development for improving the introduction of RNAi-based constructs into intracellular environment^40^. A good number of RNAi-based therapeutics are currently in clinical trials and positive results from these trials have also facilitated strengthening of further attempts to develop clinically relevant RNAi therapies^41^. Application of RNAi therapeutics in combination with potential vaccine candidates might be explored to induce long-lasting protection in livestock population. However, the availability of huge amount of experimental data from rigorous research is required to further access the potentiality of RNAi in the management of FMD outbreaks.

## Supplementary Information

Supplementary Information.
