# Supplementary material for: Computational insights into RNAi-based therapeutics for foot and mouth disease of Bostaurus
Source: Sci Rep. 2020 Dec 9;10:21593. doi: 10.1038/s41598-020-78541-6 (PMC7725835; doi:10.1038/s41598-020-78541-6)
Supplement: Supplementary file 1 — Supplementary Information. [file 41598_2020_78541_MOESM1_ESM.doc]

**Computational insights into RNAi-based therapeutics for foot and mouth disease of *Bos taurus***

Tanmaya Kumar Sahu, Anoop Kishor Singh Gurjar, Prabina Kumar Meher, Cini Varghese, Sudeep Marwaha, Govind Pratap Rao, Anil Rai, Neha Guleria, Suresh H. Basagoudanavar, Aniket Sanyal, Atmakuri Ramakrishna Rao

---------------------------------------------------------------------------------------------------------------------

Supplementary Table 1. List of 60 simulated nucleotide sequences (SNSs) with their targets and similarity with miRBase miRNAs.

| **Sl. No.** | **ID** | **Sequence** | **Target Accession** | **Similarity with miRNAs with % identity** |
| --- | --- | --- | --- | --- |
| 1 | Sim_1119 | CAGAGCAAGCGACGCCGCAATCCAC | FV536913.1 | No significant similarity with any miRNA |
| 2 | Sim_1120 | CAGAGCAAGCGACGCCGCAATCCAC | FV536925.1 | No significant similarity with any miRNA |
| 3 | Sim_1121 | CAGAGCAAGCGACGCCGCAATCCAC | FV536929.1 | No significant similarity with any miRNA |
| 4 | Sim_1122 | GAGAATTTGGAATTTCTCGAGGCTT | FV536913.1 | No significant similarity with any miRNA |
| 5 | Sim_1123 | GAGAATTTGGAATTTCTCGAGGCTT | FV536925.1 | No significant similarity with any miRNA |
| 6 | Sim_1124 | GAGAATTTGGAATTTCTCGAGGCTT | FV536929.1 | No significant similarity with any miRNA |
| 7 | Sim_1172 | CGCATACCAACGTCCAACGCCGTGA | FV536925.1 | No significant similarity with any miRNA |
| 8 | Sim_12328 | ACCAATCAAACCTAACCCGTGCAAA | FV536925.1 | oan-miR-125-3p (100%), dre-miR-125b-1-3p (100%), ptr-miR-557 (100%), osa-miR1429-3p (100%), hsa-miR-557 (100%) |
| 9 | Sim_12611 | GTGTCCCACACAAAAACCGATACTC | FV536913.1 | mtr-miR5296 (100%), vvi-miR3635-3p (100%) |
| 10 | Sim_14826 | GGCGCGCCTAAACAGCCACGCAACC | FV536933.1 | mdo-miR-7264-5p (100%), osa-miR2100-3p (100%) |
| 11 | Sim_16400 | CTCATCACAACCGACACTTAAACTA | FV536909.1 | mtr-miR5746 (100%), gma-miR4415b-3p (100%), gma-miR4415a-3p (100%) |
| 12 | Sim_16401 | CTCATCACAACCGACACTTAAACTA | FV536917.1 | No significant similarity with any miRNA |
| 13 | Sim_16635 | AACGCACTATAACGACACCACACTC | FV536933.1 | mmu-miR-878-3p (100%) |
| 14 | Sim_16636 | TGTAGCCAACTGCCGATTCGTCTGG | FV536933.1 | No significant similarity with any miRNA |
| 15 | Sim_19791 | TACGCACTGATTAACCTAAATCCCT | KJ999914.1 | No significant similarity with any miRNA |
| 16 | Sim_19792 | TTATGACTCGCGTAGGCGGGAGTCG | KJ999914.1 | No significant similarity with any miRNA |
| 17 | Sim_20035 | CAAGCTCTCATGAAATGAAATGCTC | FV536913.1 | No significant similarity with any miRNA |
| 18 | Sim_20036 | CAAGCTCTCATGAAATGAAATGCTC | FV536917.1 | No significant similarity with any miRNA |
| 19 | Sim_20037 | CAAGCTCTCATGAAATGAAATGCTC | FV536933.1 | No significant similarity with any miRNA |
| 20 | Sim_20038 | TGCTACGACCCACGCGTCAATCGAT | FV536913.1 | No significant similarity with any miRNA |
| 21 | Sim_20039 | TGCTACGACCCACGCGTCAATCGAT | FV536917.1 | No significant similarity with any miRNA |
| 22 | Sim_20040 | TGCTACGACCCACGCGTCAATCGAT | FV536933.1 | No significant similarity with any miRNA |
| 23 | Sim_21145 | CACCAGTTCGAGAGTAGAAGGGAAG | FV536913.1 | lja-miR7516-5p (94.12%) |
| 24 | Sim_21146 | CGCGCGAACGACACCCGCTAAACGC | FV536913.1 | No significant similarity with any miRNA |
| 25 | Sim_22522 | TGGCCGACCTTACAAAACGCACGTT | FV536925.1 | gma-miR1527 (100%), mmu-miR-6907-3p (100%) |
| 26 | Sim_22523 | TGGCCGACCTTACAAAACGCACGTT | FV536933.1 | No significant similarity with any miRNA |
| 27 | Sim_25236 | AAGGACCAACACAAAAACACTACCT | FV536909.1 | bbe-miR-2064-3p (100%), emu-let-7 (100%), egr-let-7 (100%), pma-miR-130a-5p (100%) |
| 28 | Sim_25237 | AAGGACCAACACAAAAACACTACCT | FV536917.1 | No significant similarity with any miRNA |
| 29 | Sim_25238 | AAGGACCAACACAAAAACACTACCT | FV536921.1 | No significant similarity with any miRNA |
| 30 | Sim_25239 | AAGGACCAACACAAAAACACTACCT | FV536921.1 | No significant similarity with any miRNA |
| 31 | Sim_25240 | TGAACTGTGCGCGCCGTCGCGAACC | FV536909.1 | No significant similarity with any miRNA |
| 32 | Sim_25241 | TGAACTGTGCGCGCCGTCGCGAACC | FV536917.1 | No significant similarity with any miRNA |
| 33 | Sim_25242 | TGAACTGTGCGCGCCGTCGCGAACC | FV536921.1 | No significant similarity with any miRNA |
| 34 | Sim_25243 | TGAACTGTGCGCGCCGTCGCGAACC | FV536921.1 | No significant similarity with any miRNA |
| 35 | Sim_26217 | AATGGCTCACCAACCCACTGCGTAT | FV536933.1 | gga-miR-1658-3p (92.86%) |
| 36 | Sim_26218 | TCGACTCAGCCAAACAACCATTACC | FV536933.1 | No significant similarity with any miRNA |
| 37 | Sim_27149 | CCCATTGCCCATGGTGGCTCCTGTC | FV536923.1 | No significant similarity with any miRNA |
| 38 | Sim_27150 | CCCATTGCCCATGGTGGCTCCTGTC | FV536931.1 | No significant similarity with any miRNA |
| 39 | Sim_27151 | GCCAATACCCTACGACGACGATTAT | FV536923.1 | No significant similarity with any miRNA |
| 40 | Sim_27152 | GCCAATACCCTACGACGACGATTAT | FV536931.1 | No significant similarity with any miRNA |
| 41 | Sim_29153 | AGGCGCAACACCAGCGCATCCCCGG | FV536933.1 | No significant similarity with any miRNA |
| 42 | Sim_29521 | GCAAACTACCCGTAATAATCCGACG | FV536921.1 | No significant similarity with any miRNA |
| 43 | Sim_29851 | ACGAGCAACGCCACAACCCGTGTCT | FV536913.1 | cfa-miR-8872 (100%), cre-miR1151b-3p (100%) |
| 44 | Sim_29852 | GTTTTTGCTCTTAGCATCGCCTTGT | FV536913.1 | No significant similarity with any miRNA |
| 45 | Sim_31492 | GGGTGACCATACAACCCGACATGCT | FV536925.1 | efu-let-7c (100%) |
| 46 | Sim_31906 | ATATCACAACCTGTAATAATAACCC | FV536933.1 | No significant similarity with any miRNA |
| 47 | Sim_3446 | CCCCCACAAGAACGACATCGTTCTC | FV536909.1 | No significant similarity with any miRNA |
| 48 | Sim_3447 | CCCCCACAAGAACGACATCGTTCTC | FV536921.1 | No significant similarity with any miRNA |
| 49 | Sim_39088 | CAACTCGATCCCTCCCCGTATGAAT | KF112904.1 | No significant similarity with any miRNA |
| 50 | Sim_39089 | GCACCGACCCACCGTGACCCAAGCG | KF112904.1 | No significant similarity with any miRNA |
| 51 | Sim_43762 | AAAACAAGCAAACACCAATACCAGC | FV536917.1 | bma-miR-5853 (100%), bmo-miR-3276 (100%), cel-miR-42-5p (92.86%) |
| 52 | Sim_43763 | CTGGGTACTGCCGACTTTAAGAGCC | FV536917.1 | No significant similarity with any miRNA |
| 53 | Sim_44038 | CATCTCCCAGCCACAACAGTGCTCC | FV536923.1 | pma-miR-4548 (100%), bta-miR-664b (100%), sja-miR-3489 (100%), ssc-miR-664-3p (100%) |
| 54 | Sim_46746 | ATGTACAACCACGTCAAAAAAACAT | FV536919.1 | mmu-miR-302a-5p (100%) |
| 55 | Sim_49022 | ACCTAGGCTCTTGAGCTGGAGTTAG | FV536933.1 | rno-miR-871-5p (100%) |
| 56 | Sim_49023 | TCCGGCACCAATACGCCGTAACTTA | FV536933.1 | No significant similarity with any miRNA |
| 57 | Sim_5744 | CTCCACGTCCCGGGAAGACCGGGAC | FV536913.1 | No significant similarity with any miRNA |
| 58 | Sim_5745 | TCCCAGTCAACCCAACGACCCGCAG | FV536913.1 | No significant similarity with any miRNA |
| 59 | Sim_821 | CCTGACGCCCGACAAACCAATTACC | FV536909.1 | bmo-miR-3308-5p (100%) |
| 60 | Sim_822 | CCTGACGCCCGACAAACCAATTACC | FV536917.1 | No significant similarity with any miRNA |

**
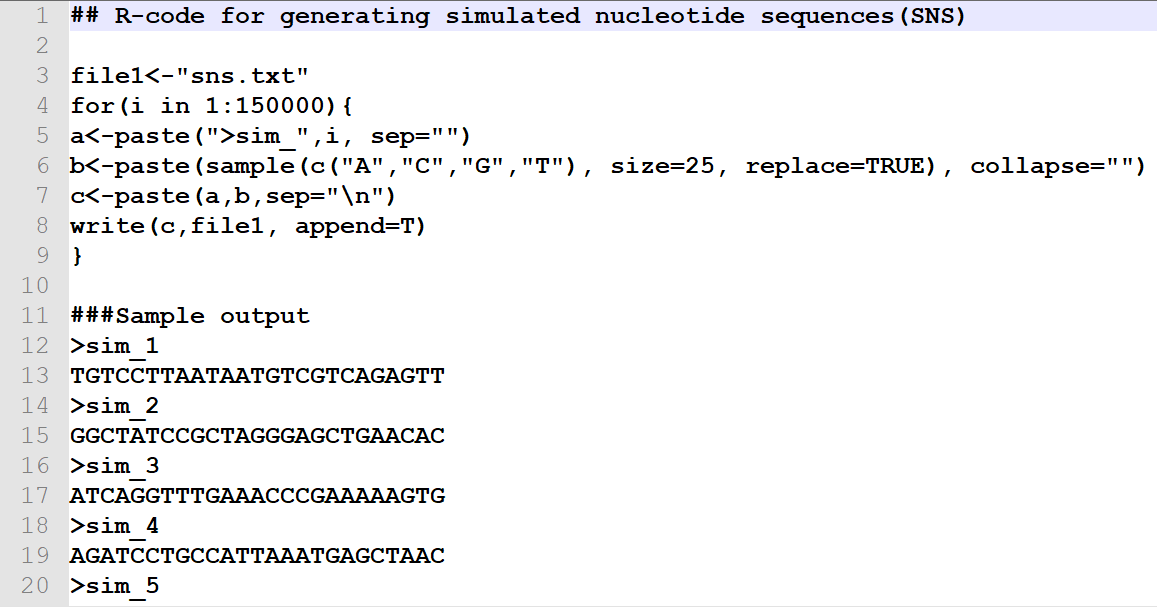
**

Supplementary Figure 1. R-code used for generating 150000 SNSs with a sample output.

**
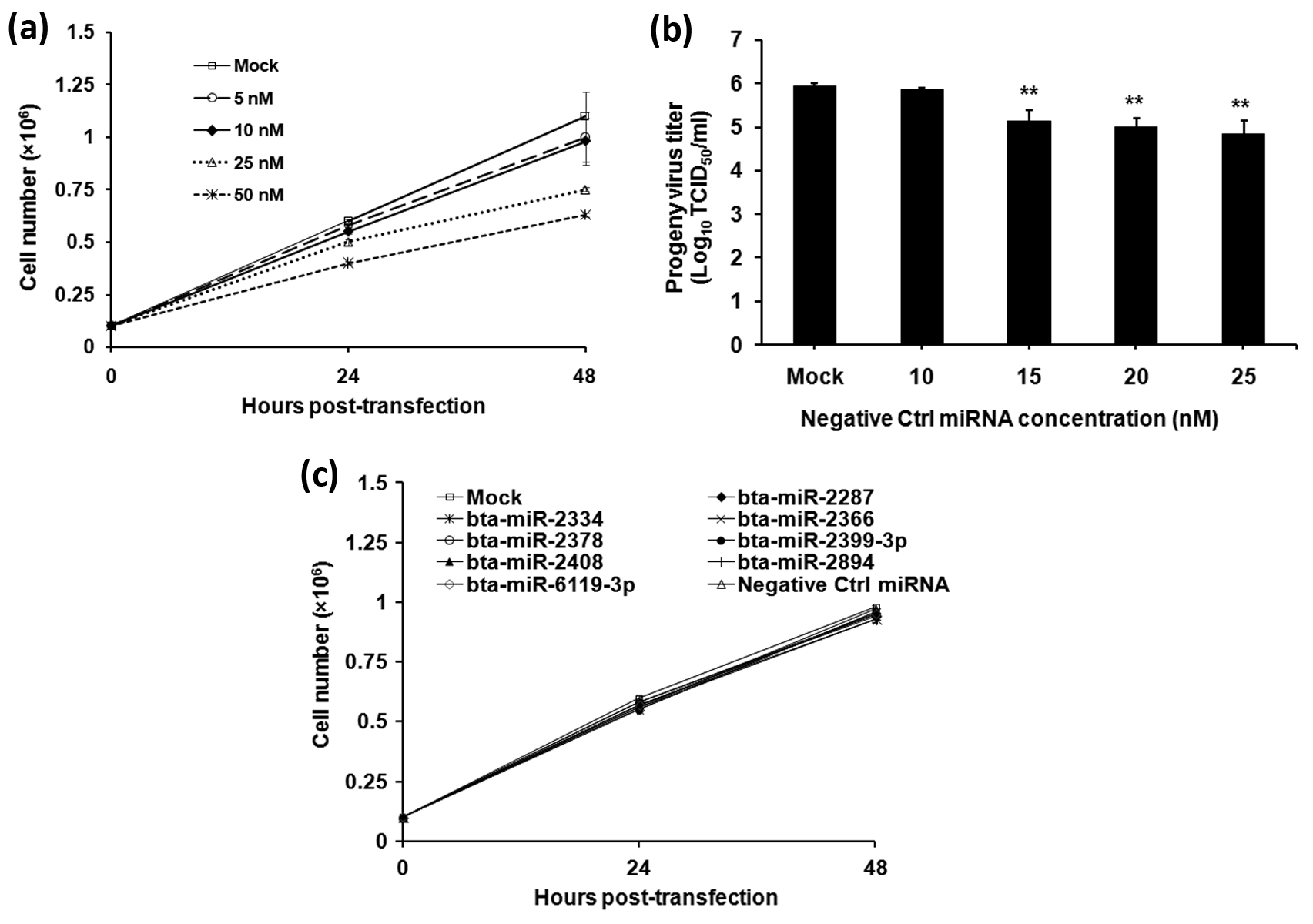
**

Supplementary Figure 2. (a) Cellular toxicity of BHK-21 cells transfected with indicated concentrations of negative control miRNA. The miRNA mimic transfection concentrations at 25 nM and 50 nM showed significant toxicity in the cells in comparison to mock control. (b) FMDV progeny yield (TCID_50_/ml) at 12 hours post infection with FMDV (0.001 MOI) in BHK-21 cells transfected with indicated concentrations of negative control miRNA in comparison with mock control for 24 hour, * *p* <0.05, ** *p* <0.01. (c) Transfection of indicated test miRNAs at 10 nM concentration showed no toxicity in the BHK-21 cells at 24 and 48 hours post-transfection. The results are expressed as "mean ± standard deviation" from three independent experiments.
